# Awareness on Girl Child Abuse Among Mothers of A Selected Community

**DOI:** 10.31729/jnma.3758

**Published:** 2018-10-31

**Authors:** Taniya Thapa, Khagi Maya Pun, Krishna Bahadur Raut, Kalpana Silwal, Rajendra Kumar Chaudhary

**Affiliations:** 1 Chitwan Medical College, School, of Nursing, Bharatpur, Chitwan, Nepal; 2 Lalitpur Nursing Campus, Sanepa, Lalitpur, Nepal; 3 Department of Emergency, Chitwan Medical College, Chitwan, Nepal; 4 Department of Obstetrics and Gynaecology, Pokhara Academy of Health Sciences, Pokhara, Nepal

**Keywords:** *Abuse*, *Awareness*, *Girl Child*, *Mothers*

## Abstract

**Introduction:**

Girl Child Abuse is physically, emotionally, sexually abusing and neglecting the girl child by depriving her of universally accepted child rights. We aim to determine the awareness on girl child abuse among mothers so that necessary awareness programs could be recommended if found unsatisfactory.

**Methods:**

A descriptive cross-sectional study was done in Sundarbazar municipality of Lamjung district among randomly selecting 288 mothers who participated voluntarily in face to face interview that used structured questionnaire from 27^th^ March to 23^rd^ April, 2016. Data was analyzed by using descriptive and inferrential statistics like frequency, percentage, mean, standard deviation, chi-square and linear by linear association.

**Results:**

The findings of the study revealed that majority of mothers 224 (77.8%) had average level of awareness regarding girl child abuse and only 21 (7.3%) had good level of awareness with mean score±SD of 45.94±9.94 (total score-76). Awareness of mother on girl child abuse was found significantly associated with age, ethnicity, educational status, type of family, age at marriage and number of children at P<0.05.

**Conclusions:**

The mothers had average level of awareness regarding girl child abuse; however, significant proportion of mothers still lacks good level of awareness. A nationwide study of such kind using qualitative tools as well as conducting awareness raising activities focusing on girl child abuse and sexual abuse in girl child is recommended.

## INTRODUCTION

Child abuse means that child is harmed, mistreated or neglected by other individual, which could be physical, sexual and emotional or ignorance among which sexual abuse is dangerous because of its immediate and upcoming effect on the child.^1^ Child abuse occurs at every socioeconomic level, across ethnic and cultural lines, within all religions and at all levels of education.^2^

A review of studies from several developing countries shows that globally a majority (80–98%) of children suffer from physical punishment at home.^3^ However, there is also a growing concern about domestic violence and related abuse of children by their mothers.^4^ It is tough to get exact data of girl child abuse because child herself is unable to expose the problem and most often caregiver abuses the child and mostly mothers tries to hide the problem.^5^ If a woman was abused in her childhood, majority of these mothers fall into the same patterns of violent childrearing they personally experienced.^6^

This study aim to find the awareness level of mother about girl child abuse in a selected community.

## METHODS

The descriptive cross-sectional study was conducted to find out the awareness on girl child abuse among mothers of Sundarbazar Municipality, Lamjung. Total of 288 mothers (using formula as n = z^2^pq/d^2^) having girl child less than 16 years residing in Sundarbazar municipality of Lamjung district were participated in the study.

Sample Size Calculation (n): z^2^pq/d^2^ where,

z= Confidence Interval at 95%, 1.96 p= Prevalence,
q= 1-p,
d= margin of error, 5%

Firstly, Sundarbazar municipality was purposively selected from Lamjung district. Then, four wards were chosen to be studied by simple random sampling (lottery method). Structured questionnaire was developed from related review of literatures on awareness on girl child abuse and the analysis of awareness level was done as:

Poor level of awareness- Score less than 38 (< 50%). Average level of awareness- Score 38 - 57 (50 - 75%) Good level of awareness- Score more than 57 (>75%)^11^

The content validity was maintained through consultation with the subject expert as well as research advisor and extensive literature review. Pre-testing of the instrument was done among 28 (10% of the sample size) mothers. Reliability of the instrument was tested using Guttman Split-Half Co-efficient which gave satisfactory value of 0.868. Study was conducted after written permission from authority concerned of Lalitpur Nursing Campus, Instututional Review Board (IRB), Tribhuwan University Institute of Medicine (T.U. I.O.M), Maharajgung and district authorities. Mothers were explained about the objectives of the study and verbal and written consent was taken before starting interview. Mothers were informed that they can interrupt the interview and withdraw whenever they want or feel uneasy to answer. Confidentiality was maintained by providing privacy during face to face interview and destroying the records after the entry of data. The data was collected by researcher herself via door-to-door home visit with the help of Female and Child Health Volunteers (FCHVs) and face to face interview. Data collection was done from 27^th^ March to 23^rd^ April, 2016.

The collected data was checked for accuracy, utility and completeness. Corrections were implemented during field and central editing. After numbering each questionnaire, classification of respective categories was made for simplification. The obtained data was entered and analyzed in Statistical Package for Social Sciences (SPSS) version 20 using percentage and measure of central tendency as uni-variate analysis while association between awareness scores and selected demographic variables were found out using Chi-square and linear by linear association test as bi-variate analysis.

## RESULTS

The mothers interviewed in Sundarbazar municipality, Lamjung belonged to age group 30–39 yrs 123 (42.7%) followed by 20–29 yrs 121 (42%) with mean age±SD of 32.32±3.6. While comparing the ethnicity status, Janajatis were found to be the highest among them 104 (36.1%) followed by Chhetri 76 (26.4%). Majority of mothers 251 (87.2%) were found to be literate. It further shows that majority of mothers were found to be married at age ranging 20–29 yrs 154 (53.5%) and majority of mothers had children less than three 225 (78.1%).

Most of the mothers 211 (73.5%) had answered neglect as one of the types of girl child abuse followed by sexual abuse against girl child 185 (64.5%). Least answered response was emotional abuse 168 (58.5%) as type of girl child abuse as shown (Table 1).

**Table 1.** Mother's Awareness on types of Girl Child Abuse.

Variables	n (%)
Neglect against girl child	211 (73.5)
Sexual abuse against girl child	185 (64.5)
Emotional abuse against girl child	168 (58.5)
Physical abuse against girl child	166 (57.8)

Most of the respondents 205 (71.7%) were aware of teaching child about privacy of body parts as prevention of abuse among girl child. The least aware preventive measure was found to be listening when child is trying to tell something which seems hard for her to talk about 161 (56.3%) (Table 2).

**Table 2.** Mother's Awareness on Methods of Preventing Sexual abuse in Girl Child.

Variables^*^	n (%)
Teach your child about privacy of body parts	205 (71.7)
Make sure you know where and with whom your child is spending her time	195 (68.2)
Not to keep child with other adults alone for long time/overnight	183 (64)
Give your child enough time so that she won't seek attention of other adults	172 (60.1)
Never leaving child alone	170 (59.4)
Listen when your child tries to tell you something which seems hard for her to talk about it	161 (56.3)

* *Multiple Responses*

The mother's level of awareness on girl child abuse which shows 224 (77.8%) had average level of awareness, 43 (14.9%) had poor level of awareness as shown (Table 3).

**Table 3.** Mother's Level of Awareness on Girl Child Abuse.

Level of Awareness	n (%)
Poor	43 (14.9)
Average	224 (77.8)
Good	21 (7.3)

*Mean Score* ± *SD = 45.94* ± *9.94*

A significant association was observed between their level of knowledge and selected demographic variables that include age, ethnicity, educational status, type of family, age at marriage and number of children since the P value is less than 0.05 (Table 4).

**Table 4.** Association of Awareness Level of Mothers with Age, Ethnicity and Religion.

Level of Awareness					
Variables	Poor	Average	Good	Type of association	P
Age				Linear-by-Linear	
20–29 yrs	3 (2.5%)	108 (89.3%)	10 (8.3%)	28.475	0.000*
30–39 yrs	21 (17.1%)	93 (75.6%)	9 (7.3%)		
40–49 yrs	19 (43.2%)	23 (52.3%)	2 (4.5%)		
Ethnicity				Linear-by-Linear	
Janajati	13 (12.5%)	82 (78.8%)	9 (8.7%)	2.285	0.039*
Chhetri	12 (15.8%)	63 (82.9%)	1 (1.3%)		
Brahmin	7 (10.4%)	52 (77.6%)	8 (11.9%)		
Dalit	11 (26.8%)	27 (65.9%)	3 (7.3%)		
Educational Status				Linear-by-Linear	
Literate	14 (5.6%)	216 (86.1%)	21 (7.3%)	97.837	0.000^*^
Illiterate	29 (78.4%)	8 (21.6%)	0 (0%)		
Type of Family				Pearson Chi-Square	
Single	13 (9.3%)	116 (82.9%)	11 (7.9%)	6.837	0.033^*^
Joint	30 (20.3%)	108 (73%)	21 (7.3%)		
Age at Marriage				Pearson Chi-Square	
< 20 yrs	28 (20.9%)	94 (70.1%)	12 (9%)	8.798	0.012^*^
20–29 yrs	15 (9.7%)	130 (84.4%)	9 (5.8%)		
No. of Children				Linear-by-Linear	
< 3	23 (10.2%)	184 (81.8%)	18 (8%)	13.898	0.000^*^
≥ 3	20 (31.7%)	40 (63.5%)	3 (4.8%)		

* *Significant (P<0.05)*

## DISCUSSION

The Convention on the Rights of the Child (CRC) recognizes the individual human rights of children virtually adopted by all the countries including Nepal covering the four broad categories of child rights: survival, development, protection and participation.^7^ Since the Cairo International Conference on Population and Development in 1994, and the Beijing Conference on Women and the Girl Child in 1995, gender equality has been a priority area of demographic research. According to United Nations, International Day of Girl Child 2015 (Vision for 2030) aims to promote zero tolerance against physical, mental, and sexual harassment and violence.^8^ Uncaring parents with inadequate awareness about different forms of child abuses was found as the reason why children are neglected and thus exposed to different forms of abuse as perceived by the childre.^9^

In this study, mean age±SD of mothers was 32.32±3.6 yrs which is similar to the study by Gurung and Bhattarai^10^ among 95 parents of Kathmandu valley with median age of 30 yrs (range 19–55 yrs). In this study, 88% were literate and 48.6% of them lived in nuclear family. This finding has similarities with the study in which 84.2% of the respondents were literate but in contrast, 80% of them lived in nuclear family.^10^

More than half of mothers were aware of physical abuse (57.8%) and emotional abuse (58.5%) as types of girl child abuse and even higher than these; neglect (73.5%) and sexual abuse (64.5%) were told as types of girl child abuse. Most of the mothers (71.7%) were aware of teaching child about the privacy of body parts for preventing sexual abuse in this study. In this study, 77.8 % of the mothers had average level of awareness, 14.9 had poor level of awareness and only 7.3% had good level of awareness with mean±SD of 45.94 ± 9.94 out of total 76 score. Similar study shows majority i.e. 50.53% of the participants in the study were found to have good knowledge on overall child abuse and poor knowledge among mothers was found least that was 1.1%.^10^ From the above comparison we can conclude that respondents having good level of awareness were found more in the study by Gurung & Bhattarai than compared to this study which may be due to more backwardness of the area as well as unavailability of adequate informational sources.

The findings of this study shows association between awareness on girl child abuse and few demographic variables i.e. age of respondents, their age at marriage, ethnicity, type of family, educational status and number of children. It is similar with the findings of Gurung & Bhattarai^10^ which depicts significant association of awareness level and between age group of parents but no association with educational status at 5% level of significance. Another research which further indicated that mother's education and family size are significant determinants of child abuse and its awareness as compared to the socio-economic status and father's education which are non-significant.^11^

## CONCLUSIONS

Majority of mothers residing in Sundarbazar municipality had average level of awareness on girl child abuse but good level of awareness was found still very low which gives a serious matter among the girl child. This study provided a new perspective to the underexplored area of child abuse and opened the door for a large scale study to get the actual scenario of its awareness as well as its occurrence throughout the nation.

## Conflict of Interest

**None.**
